# Supplementary material for: An improved control strategy of power-frequency characteristics of virtual synchronous generator
Source: PLoS One. 2026 May 6;21(5):e0348794. doi: 10.1371/journal.pone.0348794 (PMC13148683; doi:10.1371/journal.pone.0348794)
Supplement: Attachment — Submitted filename: point-by-point response.doc [file pone.0348794.s002.doc]

**Dear Editors:**

Thank you for your comments concerning our manuscript entitled “An improved control strategy of power-frequency characteristics of virtual synchronous generator”. We have studied comments carefully and have made correction which we hope meet with approval. The correction in the paper and the responds to your comments are as following:

Responds to the comments:

**Academic editor:**

The readability of the paper needs to be further improved, the quality of the figures should be enhanced, and the descriptions of some terms should be kept consistent.

**Author Response:**

According to the editor's requirements, the author has standardized and corrected the relevant technical terms in the paper, and has also improved the quality of the figures and tables.

**Additional Editor Comments:**

The paper needs to be strengthened in the following aspects:

1. The paper should provide the tuning method of control parameters. From the perspective of controller design, the proposed method relies too heavily on the model. When the model parameter identification is inaccurate, can the effectiveness of the algorithm be guaranteed?

**Author Response:**

In the revised manuscript, the author added the closed-loop pole distribution diagram of the active power response closed transfer function G_2_(s) of the traditional virtual synchronous generator, as shown in Fig 6 in the revised manuscript. By using Fig 6, the influence of the virtual rotational inertia J and damping D on the power characteristics of the VSG was further analyzed. This provided a more in-depth explanation of the coupling mechanism between J and D, as well as the mutually exclusive issue between the dynamic adjustment characteristics and steady-state performance of the VSG brought about by this coupling.

In the revised manuscript, the author has added the closed-loop pole distribution diagram of the improved VSG active power response closed-loop transfer function G_B2_n_ (s) (as shown in Fig 12 and Fig 13 in the revised manuscript). Fig 12 shows the pole variation trajectory of G_B2_n_(s) when the time constant *T* of the inertia correction link is changed, and Fig 13 shows the pole variation trajectory of G_B2_n_(s) when the control parameters *C* of the lead differential link are changed. By combining Fig 12 and Fig 13, the author has supplemented and explained how to adjust the control parameters T and C to improve the operating characteristics of the VSG.

2. The stability analysis needs to be supplemented. According to the experimental data, obvious chattering appears in the control error. What is the cause of this phenomenon?

**Author Response:**

In the revised manuscript, the author conducted a supplementary analysis on the stability issue of the improved VSG by integrating the newly added Fig 12 and Fig 13.

When the virtual inertia *J* of the traditional VSG is large or its virtual damping *D* is small, it can cause system oscillation. In the improved VSG, the chattering phenomenon that appears in the control error can be suppressed by increasing the control parameter *C* of the lead differential control loop. The experimental results in Fig 17 and Fig 19 of the revised manuscript show that increasing the control parameter *C* of the lead differential loop can significantly reduce the frequency and power fluctuations of the improved VSG.

3. lease supplement the diagrams of experimental equipment and describe the experimental setup in detail.

**Author Response:**

In the revised manuscript, the author added Figure 17 and provided additional explanations for the experimental setup.

**Reviewer #1:**

This paper proposes an improved control strategy for VSG to address the regulation contradiction between virtual inertia and damping. The topic is interesting. However, the contributions of this work appear to lack novelty. The reviewer offers the following comments for consideration:

1. In the introduction, some relevant studies on power oscillation damping methods for VSGs from the last five years are missing. For instance, virtual impedance control, additional damping control, and mutual damping control can also reduce control overshoots without affecting steady-state performance.

**Author Response:**

According to the reviewer’s comment, the author supplemented discussion on the recent five-year research progress regarding VSG decoupling control in the third paragraph of the introduction, and added seven references (References [20]- [26]).

2. The analysis in grid-connected mode assumes a strong grid with infinite capacity ( ωg = ω0 ). In practical applications, grid strength may vary and can affect the system's dynamic response. The authors are encouraged to analyze and evaluate the robustness of the proposed control strategy under varying grid strength conditions.

**Author Response:**

The improved VSG proposed in the paper mainly aims to enhance the degrees of freedom of the traditional VSG, and can effectively achieve the decoupling control of the virtual inertia *J* and damping *D* of the VSG. In the revised manuscript, the author supplements the pole distribution diagram of the closed-loop transfer function G_B2_n_(s) of the active power response of the improved VSG (as shown in Fig 12 and Fig 13 in the revised manuscript), and use the zero-pole distribution diagram to supplement the discussion on the influencing factors of the proposed decoupling control strategy. If the application scenario changes to a smaller-capacity weak grid where the frequency stability of the grid is relatively low, the improved VSG needs to have stronger frequency support capabilities. We will continue to conduct research in this area in the future.

3. The manuscript only considers a single VSG. It would be valuable to investigate the performance and scalability of the proposed control strategy when applied to systems with multiple VSGs operating in parallel.

**Author Response:**

The VSG virtual inertia *J* and damping *D* decoupling control strategy proposed in the paper is applicable to both off-grid or grid-connected operation control of a single VSG, as well as off-grid or grid-connected operation control of multiple VSGs connected in parallel. The off-grid or grid-connected operation of multiple VSGs also involves the power distribution issue among the VSGs, which is a broader research field. We are very grateful to the reviewers for providing us with new research ideas. We will further explore this issue in our subsequent research work.

4. The superiority of the proposed control method requires further validation. Specifically, its performance should be compared with state-of-the-art power oscillation damping methods to demonstrate its advantages, at least by providing a comparative analysis with existing adaptive methods (e.g., VSG with adaptively tuned J and D).

**Author Response:**

The VSG with adaptive adjustment of *J* and *D* does not fundamentally solve the defect of coupling between *J* and *D*. The improved VSG control strategy proposed in this paper can achieve decoupling control of *J* and *D*. In the revised manuscript, the authors compare the VSG decoupling control strategy proposed in this paper with other decoupling control strategies in the third and fourth paragraphs of the introduction.

**Reviewer #2:**

The author proposed a method to supress power oscillation of virtual synchronous generator (VSG) in the transition process. The mathematical model of VSG, control algorithm and closed-loop transfer function of active power to frequency fluctuation are developed. The simulation results show that the proposed method is good to supress the power oscillation.
Hence, this manuscript can be accepted.
