# Supplementary material for: An improved control strategy of power-frequency characteristics of virtual synchronous generator
Source: PLoS One. 2026 May 6;21(5):e0348794. doi: 10.1371/journal.pone.0348794 (PMC13148683; doi:10.1371/journal.pone.0348794)
Supplement: Attachment — Submitted filename: Response to the reviewer.doc [file pone.0348794.s003.doc]

**Dear Editors:**

Thank you for your comments concerning our manuscript entitled “An improved control strategy of power-frequency characteristics of virtual synchronous generator”. We have studied comments carefully and have made correction which we hope meet with approval. The correction in the paper and the responds to your comments are as following:

Responds to the comments:

**Academic editor:**

The readability of the manuscript needs to be further improved to enhance its appeal.

**Author Response:**

According to the editor's requirement, the author polished the entire manuscript to enhance its readability. The revised content can be found in the blue fields of the revised manuscript.

**Additional Editor Comments:**

The paper still has certain deficiencies in the analysis of research status and the description of experimental equipment. It is suggested to supplement the analysis of research status in the past three years and provide a detailed introduction to the experimental equipment.

**Author Response:**

In the revised manuscript, the author updated and replaced references 1, 5, 7, and added references 18, 21, 29, 30, 31. In the introduction, the author also conducted supplementary analysis of the research status in the past three years. The modified and supplementary content can be found in the red fields of the introduction and references.

According to the editor's requirement, the author provides additional descriptions of the experimental equipment in the revised manuscript. The supplementary content can be found in the red fields of "Experiment verification".
